# Alcoholic and Arsenical Neuritis

**Published:** 1903-08-15

**Authors:** 


### ALCOHOLIC AND ARSENICAL NEURITIS.

Dr. Reynolds[1] again directs attention to the distinction which should be drawn between the neuritis due to chronic arsenical poisoning and that due to alcohol. It is probable, he thinks, that many of the cases of so-called alcoholic neuritis are in reality due to arsenic, apart altogether from the recent epidemic in the Northern and Midland Counties. As he has previously shown, impure glucose is not the only source of arsenic in beer. Malt, especially when prepared by the use of certain cokes, may be contaminated, and beer prepared from such malt may contain appreciable quantities of arsenic, and so produce symptoms of neuritis in those who drink it. The symptoms of genuine alcoholic neuritis as it occurs in spirit-drinkers are, Dr. Reynolds believes, different from those of arsenical neuritis. In the former there is some loss o' power in the legs and arms, with absent knee-jerks, paræsthesia of the feet and hands, only slight tenderness on pressure of the muscles, very little atrophy, no loss of memory and no œdema. Such cases are rare. In the other and more common kind, which is due to arsenical poisoning, much paralysis and atrophy of the legs and arms are present, together with loss of knee-jerks, great tenderness on pressure of the muscles, and peculiar and typical loss of memory for time and place, and often œdema of the legs from dilatation of the heart. This was the type commonly met with during the recent epidemic, various skin lesions also being met with, including pigmentation, keratosis, herpes, and erythema. During the last two years, in which period Manchester beers have contained not more than $\frac{1}{100}$ grain of arsenic per gallon, cases showing these symptoms have practically disappeared from the city, up to about three months ago. During the last two months, however, Dr. Reynolds has seen six cases; he therefore has had analysed samples of beer from two different breweries with which some of the patients had been supplied. Both these samples were found to contain arsenic in quantities certainly greater than $\frac{1}{50}$ gr. per gallon, and possibly as much as $\frac{1}{25}$ gr. per gallon. The source of this arsenical contamination is not clear, but the fact of its discovery coincidentally with the appearance of six cases of neuritis certainly goes to support the view which Dr. Reynolds maintains, that many of the so-called cases of alcoholic neuritis are in reality due to poisoning by arsenic.

[1] British Medical Journal, July 25th.

